# Social Milieu Oriented Routing: A New Dimension to Enhance Network Security in WSNs

**DOI:** 10.3390/s16020247

**Published:** 2016-02-19

**Authors:** Lianggui Liu, Li Chen, Huiling Jia

**Affiliations:** School of Information Science and Technology, Zhejiang Sci-Tech University, Hangzhou 310018, China; kunjiande@163.com (L.C.); wannahealthy@sohu.com (H.J.)

**Keywords:** social milieu oriented routing, quality of trust, quantum annealing, quantum tunneling, WSNs

## Abstract

In large-scale wireless sensor networks (WSNs), in order to enhance network security, it is crucial for a trustor node to perform social milieu oriented routing to a target a trustee node to carry out trust evaluation. This challenging social milieu oriented routing with more than one end-to-end Quality of Trust (QoT) constraint has proved to be NP-complete. Heuristic algorithms with polynomial and pseudo-polynomial-time complexities are often used to deal with this challenging problem. However, existing solutions cannot guarantee the efficiency of searching; that is, they can hardly avoid obtaining partial optimal solutions during a searching process. Quantum annealing (QA) uses delocalization and tunneling to avoid falling into local minima without sacrificing execution time. This has been proven a promising way to many optimization problems in recently published literatures. In this paper, for the first time, with the help of a novel approach, that is, configuration path-integral Monte Carlo (CPIMC) simulations, a QA-based optimal social trust path (QA_OSTP) selection algorithm is applied to the extraction of the optimal social trust path in large-scale WSNs. Extensive experiments have been conducted, and the experiment results demonstrate that QA_OSTP outperforms its heuristic opponents.

## 1. Introduction

Wireless sensor networks (WSNs) [1] have prevailed in many areas, such as battlefield sensing, environmental monitoring, disaster handling, and traffic control, and have gained much attention from the world in recent years. In WSNs, sensor nodes with limited communication capabilities, less computation power, and less memory are deployed in the specific environment. Due to the constraints, conventional routing protocols are designed for resource-limited self-organized WSNs, which have been widely explored in recent years [2,3,4,5,6,7,8,9]. Trust, an important social concept in all human interactions, has been proven to be a promising way to resolve the security issues raised by distributed collaborations. Recent progress in the area of social networks study shows that establishing trust in WSNs yields many benefits, such as giving nodes incentives to cooperate in packet forwarding, providing corresponding access control, and making the traditional security services more robust and reliable [10], and to the end, greatly enhance network performance in WSNs. However, few existing routing protocols take trust into consideration. On the one hand, trust establishment requires an evaluation of the trustworthiness of a trustee node along a certain trust path from a trustor node to the trustee node [11]. However, there are usually many trust paths between one trustor and trustee pair in large scale WSNs. On the other hand, mainstream trust research in wireless networks, including WSNs, mainly focuses on trust or reputation management without differentiating between trust between two nodes and recommendation from third party nodes. It has been proved that it would be better to regard trust and recommendation as two different independent Quality of Trust (QoT) attributes to reflect the real-world situation better [11]. Thus, a challenging problem is which the social trust path is the optimal one that can yield the most trustworthy evaluation result with two different independent end-to-end QoT constraints in large scale WSNs. This optimal social trust path (OSTP) selection problem subject to more than one end-to-end QoT constraint has been proved to be NP-complete [11]. Since there may be over thousands of paths between a pair of trustor and trustee nodes in large scale WSNs, evaluating the trustworthiness of the target trustee node along all these social trust paths requires a large amount of computation time [12]. To make this problem practically solvable, we propose an efficient and effective approximation algorithm, called the QA-based optimal social trust path (QA_OSTP) selection algorithm. Our main contributions are:

1. For the first time, we map the aforementioned OSTP selection problem in large scale WSNs onto a transverse Ising spin glass (TISG) model. Then, we utilize configuration path-integral Monte Carlo (CPIMC) technology [13] with the aid of quantum-classical mapping and Jarzynski equality to perform the practical implementation of quantum annealing (QA) and solve the time-dependent Schrödinger equation in a very large and exponentially growing Hilbert space.
2. We carry out the performance evaluation of the newly constructed QA_OSTP and compare it with the referenced method based on extensive experiments. The experimental results demonstrate the effectiveness of QA_OSTP and prove that QA_OSTP has better performance in terms of the utility of the selected social trust path and execution time than its counterpart does.

The rest of this paper is organized as follows. Section 2 summarizes related work in literature. In Section 3, the novel QA-based OSTP selection algorithm (QA_OSTP) is presented, and efficiency concerns are addressed. Section 4 contains experimental results and analysis, which show that QA_OSTP outperforms state-of-the-art heuristic algorithm**s** significantly. Finally, we conclude this paper in Section 5 with a summary and future research direction.

## 2. Related Work

The OSTP selection with multiple end-to-end QoT constraints can be modeled as the classical multi-constrained optimal path (MCOP) selection problem [11]. In this section, we review some existing MCOP and OSTP selection algorithms.

Korkmaz *et al.* [14] first proposed an approximation algorithm H_MCOP to determine a feasible path that satisfies a set of constraints while maintaining high utilization of network resources. H_MCOP is a promising method. However, when the possible path with the maximal utility is not a feasible solution, H_MCOP will stop searching, which will lead to the failure of finding a near-optimal solution and sometimes returning an infeasible one even when a feasible solution exists.

Yu *et al.* [15] studied the problem of service selection with multiple QoS constraints and proposed an approximation algorithm, MCSP-K based on H_MCOP. However, in the service candidate graph therein, there is a link between any two nodes in adjacent service sets. If this requirement cannot be satisfied in a network, MCSP_K will search all the paths from a source node to each intermediate, which will lead the time complexity to be exponential. Thus, this algorithm does not fit for large scale WSNs.

In [16], N. Alhadad *et al.* took the entities of distributed systems and the relationships between them into consideration and raised two issues. The first one is how to formalize the entities that compose a system and their relationships for a particular activity. The other one is how to evaluate trust in a system for this activity. They proposed answers to both questions. On the one hand, they proposed SocioPath, a meta-model based on first order logic, which allows one to model a system considering entities of the social and digital worlds and their relationships. On the other hand, the authors proposed two approaches to evaluate trust systems, namely, SocioTrust and SubjectiveTrust. The former is based on probability theory to evaluate users’ trust in systems for a given activity. The latter is based on subjective logic to take into account uncertainty in trust values. However, their methods only take into account one parameter (*i.e.*, trust) in their evaluation and cannot be applied to deal with the NP-complete problem mentioned in this paper.

Most of the existing work considered only indirect trust computation methods and integrated trust computation methods into comprehensive indirect computation to calculate the indirect trust for indirectly linked users. Unlike the previous work, M. Li *et al.* [17] proposed a trust evaluation scheme for user relationships (links) in a social network. Their approach comprises two aspects: the reliability degree measuring the trustworthiness of the link and the strength degree for evaluating the closeness between users. A trust calculation method was given, including direct trust for directly linked users and indirect trust for indirectly linked users, which is established based on the comment factor, the forwarding factor, and the approving factor. Then a link strength evaluation method was proposed to determine the trustworthiness of direct and indirect links between users considering comment stability, mutual trust, interaction frequency, and common neighbors and community similarity. In the end, they designed a link trust evaluation algorithm based on the link trust matrix synthesizing the reliability and strength of links. Unfortunately, similarly to N. Alhadad *et al.*’s work, their work cannot be used in the context of our paper.

G. Liu. *et al.* [11] developed a novel efficient heuristic algorithm MFPB_HOSTP for OSTP selection in complex social networks, where multiple backward local social trust paths (BLPs) are identified and concatenated with one Forward Local Path (FLP), forming multiple foreseen paths. MFPB_HOSTP is one of the most promising algorithms in solving the OSTP selection problem as it outperforms prior exiting algorithms in both efficiency and the quality of delivered solutions. However, due to its searching characters, it cannot avoid obtaining partial optimal solutions during the searching process that will lead to the failure of guaranteeing the efficiency of searching.

Recently, in order to decrease the convergence time of the previous algorithm, G. Liu. *et al.* proposed a novel algorithm named T-MONTE-K [18] by combining the Monte Carlo method and their optimized search strategies. It shows that T-MONTE-K is an effective and efficient approximation algorithm, which considers both the QoSTP constraints and the path utility, thus avoiding lots of unnecessary probing of infeasible paths. The results of extensive experiments conducted based on a real-world dataset demonstrate that T-MONTE-K can outperform the existing state-of-the-art algorithm MONTE_K.

## 3. Algorithm Description

### 3.1. QoT Parameters Aggregation Rules and Related Definitions

In this paper, we take two independent QoT parameters, that is, trust and recommendation into consideration. We first define trustor node, target trustee node and intermediate participant in WSNs while performing social milieu oriented routing. Then, definitions of these two QoT parameters are given and related aggregation rules are described in detail.

**Definition** **1.**  *Trustor node is a type of participant in WSNs, who is the invoker of social milieu oriented routing and the performer of the aforementioned QA_OSTP algorithm*.

**Definition** **2.**  *Target trustee node is a type of participant in WSNs, who is the destination of the messages sent by the trustor node. Moreover, its trustworthiness can be represented more comprehensively based on QA_OSTP algorithm*.

**Definition** **3.**  *Intermediate participant is a type of participant in WSNs along the social trust path between the trustor node and the target trustee node, with whose help the trustor node can find his optimal social trust path to the target trustee node before invoking the social milieu oriented routing*.

In human societies, trust is a complex topic subject to previous mutual interactions between two peer parties. Many different trust definitions have been proposed addressing people’s different concerns [11]. In the context of this paper, trust between two participants in WSNs can be defined as follows.

**Definition** **4.**  *Trust is the belief showing to what degree a participant will accept its neighbor participants’ recommendation based on their past mutual interactions*. $T_{AB}\in \left[0,1\right]$ *denotes trust value that participant A assigns to participant B. When T_AB_= 0, it means that A does not trust B at all and when T_AB_ = 1, it denotes that participant A completely trust B.*

The trust value between a trustor node and the target trustee node along a social trust path can be aggregated based on the trust transitivity rule [11]. Given there are n participants *a*_1_,…,*a_n_* in order along a social trust path *p*(*a*_1_,…,*a_n_*) between the trustor node and the target trustee node, we can compute the aggregated trust value using:

(1) $$T_{p(a_{1},...,a_{n})}=\underset{\left(a_{i},a_{i+1})\in p(a_{1},...,a_{n}\right)}{\Pi }T_{a_{i}a_{i+1}}$$

Research in social psychology shows that, in the same society, the recommendation of a third party node does not decay with the increase of the number of transitivity hops [19]. From this point of view, recommendations from the third party node and its aggregation rule can be defined as follows.

**Definition** **5.**  R∈[0,1] *is the recommendation of a third party node in WSNs. R = 0indicates that the third party node has no knowledge of the trustee node. R = 1indicates that the third party node completely knows about the trustee node.*

Due to the fact that the recommendation of a participant does not decay with the increasing of transitivity hops, the recommendation value along a certain social trust path $p(a_{1},...\;a_{n})$ in WSNs can be aggregated by

(2) $$R_{p(a_{1},...a_{n})}=\frac{\sum_{i=2}^{n-1}R_{a_{i}}}{n-2}$$

Then the utility is the measurement of the trustworthiness of a social trust path, which can be defined by:

(3) $$F_{p(a_{1},...a_{n})}=w_{T}\times T_{p(a_{1},...a_{n})}+w_{R}\times R_{p(a_{1},...a_{n})}$$

where *p*(*a*_1_,…,*a_n_*) is a social trust path with *n* participants *a*_1_,…,*a_n_*, $w_{T}$ and $w_{R}$ are the weights of the aggregated trust value and the recommendation value along a social trust path between a trustor node and the target trustee node; $0<w_{T},w_{R}<1$ and $w_{T}+w_{R}=1$.

The goal of the selection of OSTP between thetrustor node and the target trustee node in WSNs is to select the optimal path undertwoQoT parameters constraints, which yields the best utility with the criteria defined by the trustor node.

### 3.2. Quantum Annealing (QA)

In statistical mechanics, a physical process called annealing is often performed in order to relax the system to the state with the minimum energy. In the basic form of simulated annealing (SA), it first generates an initial solution as the current feasible solution using the Metropolis algorithm [20]. Then, another solution is selected in the neighborhood of the current solution and replaces the current solution with the new one with the following transition probability given by the Metropolis criterion. With the temperature decreasing, only the better deterioration configuration can be accepted. Simulated annealing is a particularly promising minimization technique. It has, for example, proved effective in finding the global minimum of multidimensional functions having large numbers of local minima [21]. However, it is worth mentioning that introducing thermal fluctuation is not the only way to perform annealing. QA depends on quantum fluctuation instead [22]. A prominent advantage of quantum fluctuation over thermal fluctuation originates from the fundamental property of the quantum theory, namely, the possibility of tunneling through classically impenetrable potential barriers between energy valleys. Consequently, methods of quantum search, in principle, could be more efficient than the classical search methods [23]. Furthermore, the effect of quantum tunneling is shown to be crucial for solving many computationally difficult problems, including the class of non-deterministic polynomial time problems. A practical implementation of QA will need to solve the time-dependent Schrödinger equation in a very large and exponentially growing Hilbert space, which can only rely on a robust quantum computer. Recent research in this area has been carried out by the configuration path-integral Monte Carlo (CPIMC) approach [13] using quantum-classical mapping with the aid of a Suzuki-Trotter transformation [24,25,26], inspired from which we apply QA to the OSTP selection in WSNs. To the best of our knowledge, this is the first application of QA to this problem and experiment results show QA based searching algorithm has better performance than its heuristic opponent does.

### 3.3. Problem Representation

First, some assumptions are given as follows to eliminate some secondary factors, which will increase unnecessary complexity and may influence the performance of the WSNs:

**Assumption** **1.**  *Before searching the OSTP, the values of trust and the recommendation of third party nodes have been already obtained through mining techniques [27]*.

**Assumption** **2.**  Since the transverse Ising spin glass (TISG) model is the simplest model in which quantum effects in a random system can and have been studied extensively and systematically [28], here we concentrate our attention only on the TISG.

**Figure 1:**
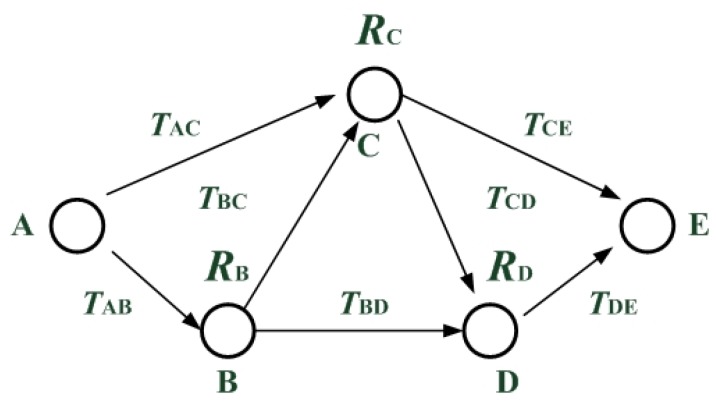
An example of WSN.

**Assumption** **3.**  *The large scale WSNs studied here are symmetric. Symmetric means that each pair of adjacent nodes, trustor and trustee, can reverse their roles without changing their trust values. For example, in Figure 1, we set* $T_{AC}=T_{CA}$.

*(i)*  Definition of Quantum Hamiltonian

In the TISG model, the total Hamiltonian can be written as:

(4) $$H_{OSTP}=H_{pot}+H_{kin}$$

where $H_{pot}$ represents the classical potential energy of a given configuration, and $H_{kin}$ is a suitable kinetic energy operator providing the necessary quantum fluctuations to escape local minima. In QA, we seek to minimize $H_{pot}$ as side effect of minimizing $H_{OSTP}$. A suitable configuration is reached if and only if $H_{pot}$ is zero.

In the TISG model, the total Hamiltonian in Equation (4) can be rewritten as:

(5) $$H_{OSTP}=H_{\text{TISG}}+H_{TF}(t)$$

where $H_{\text{TISG}}$ denotes potential energy of TISG model, and $H_{TF}(t)$ is a fictitious kinetic energy introduced typically by the time-dependent transverse field.

A TISG model consists of a set of spins, each of which can only be in one of two states. Each of these spin variables usually takes on the value of either +1 or −1, also known as an up-spin and a down-spin, respectively. Formally, for a WSNs with *N* nodes each configuration of the system (a feasible social trust path) is associated to a *N × N* matrix *U* with 0/1 entries in the following way: for each pair of the participants *m* and *n*, if the directed social trust path (an ordered sequence of participants in WSNs) goes through the link between *m* and *n*, then *U_mn_* = 1, or else *U_mn_* = 0. Here, for the sake of simplicity, we renumber the independent variables as $U_{k}(k=1,\;...,K)$ and the other dependent variables can be expressed by using *U_k_*. Then, in quantum mechanics, the quantum Hamiltonian of the OSTP selection problem can be expressed as a *K*-spins TISG model through the transformation $U_{k}\to \left(1+\sigma_{z}^{k}\right)/2$, where $\sigma_{z}^{k}$ is the Pauli matrix of qubit *k*. In addition, the possible social trust paths can be represented by different quantum states of the *K* spins:

(6) $$H_{\text{TISG}}\equiv \sum_{m=1}^{K}v_{m}\sigma_{z}^{m}+\sum_{m=1}^{K}\sum_{n=m+1}^{K}J_{mn}\sigma_{z}^{m}\sigma_{z}^{n}$$

where *m* and *n* represent two randomly selected participants, that is, two qubits in the TISG model, *ν_m_* is the off-resonance term related to *m*, $\sigma_{z}^{m}$ and $\sigma_{z}^{n}$ describe the Pauli matrix of qubit *m* and *n*, *J_mn_* denotes the spin-spin coupling between qubit *m* and *n*.

**Figure 2:**
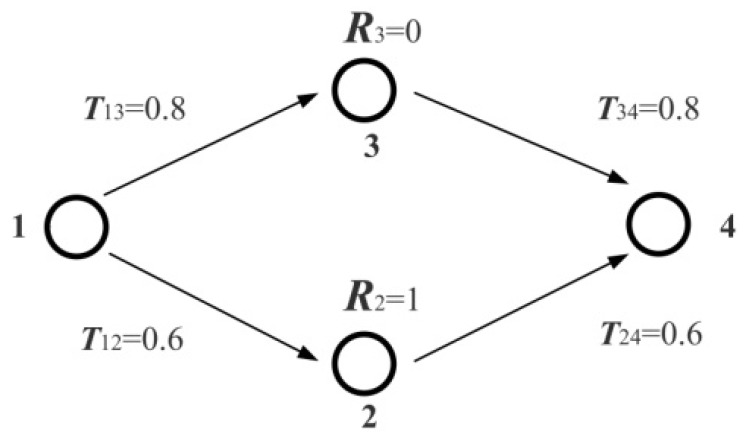
An example of WSNs (*w_T_* = 0.6, *w_R_* = 0.4).

We use Figure 2 and Table 1 as a demonstration to describe this kind of correspondence.

In Figure 2 four participants compose a WSN: Node 1 is the trustor node; Node 4 is the target trustee node; Node 2 and Node 3 are two intermediate participants. Here, weights of the two QoT parameters (trust and recommendation) are set as *w*_T_ = 0.6, *w*_R_ = 0.4, respectively. There are two possible social trust paths between trustorNode1 and trustee Node4, that is, path 1—3—4 and path 1—2—4. The utility values of these two social trust paths determined by the utility function (3) are 0.384 and 0.616, respectively. Thus, trustor Node 1 will choose path 1—2—4, not path 1—3—4, as its preferred social trust path according to the criteria specified by itself, although path 1—3—4 has a larger trust value according to the aggregation rules.

Note that the realization of quantum annealing requires introducing an artificial and adjustable quantum kinetic operator, which can provide the quantum fluctuations to escape the local minima. Moreover, the quantum annealing process is required to be slow enough to approximate the adiabatic evolution. Reasonably, the choice of *H_kin_* should encompass one important question that is determining which configurations are to become direct neighbors of a given configuration. Here we use an effective tactic named “plus-minus” [29] as a disturbance mechanism to realize a neighborhood of a given configuration. Then in TISG model, to implement QA, a fictitious kinetic energy is introduced typically by the time-dependent transverse field:

(7) $$H_{TF}(t)=H_{\text{Kin}}\equiv \Gamma (t)\sum_{i=1}^{K}S_{\langle i',i\rangle }^{+}S_{\langle j',j\rangle }^{+}S_{\langle j,i\rangle }^{-}S_{\langle j',i'\rangle }^{-}$$

where Γ(t) is the time-dependent power of the transverse field, *S* denotes the Ising spin. Each $S_{\langle i,j\rangle }^{\pm }$ flips an Ising spin variable at position (*i,j*) and at the symmetric position (*j,i*), *i.e.*, $S_{\langle i,j\rangle }^{\pm }=S_{i,j}^{\pm }S_{j,i}^{\pm }$. Consequently, we have the following form of the total quantum Hamiltonian of OSTP selection problem:

(8) $$H_{OSTP}=\sum_{i=1}^{K}v_{i}\sigma_{z}^{i}+\sum_{i=1}^{K}\sum_{j=i+1}^{K}J_{ij}\sigma_{i}^{z}\sigma_{z}^{i}\sigma_{z}^{j}+\Gamma (t)\sum_{i=1}^{K}S_{\langle i',i\rangle }^{+}S_{\langle j',j\rangle }^{+}S_{\langle j,i\rangle }^{-}S_{\langle j',i'\rangle }^{-}$$

Initially, the strength of the transverse field Γ(t) is chosen to be very large, and $H_{OSTP}$ is dominated by the third term of Equation (8). Then Γ(t) is gradually and monotonically decreased toward zero, leaving eventually only the first two terms. Note that every state of the TISG model can be described as a state vector it will evolve with time and should follow the RT Schrödinger equation.

With Γ(t) decreasing, accordingly, the state vector |ψ(t)〉 is expect to evolve from the expected initial ground state of transverse-field term Equation (5) the transverse-field l ground state of Equation (6), which is the solution of the OSTP selection problem.

Then an important issue is how slowly we should decrease Γ(t) to keep the state vector arbitrarily close to the instantaneous ground state of $H_{OSTP}$. As mentioned in Section 3.2, we will not attempt an actual Schrödinger annealing evolution of the quantum Hamiltonian due to the large Hilbert space. On the contrary, we address the quantum problem by CPIMC-based QA, where annealing will take place in the fictitious time represented by the number of CPIMC steps. However, in order to figure out the OSTP selection problem by CPIMC-based QA, a Suzuki-Trotter transformation should be performed in advance, which requires calculation of the matrix elements of an exponential operator between arbitrary configurations |ψ〉 and 〈ψ'| of the system. Moreover, since the energy gap between the ground state and the first excited state is large at the beginning, and decreases with the annealing time, hyperbolic interpolation makes the annealing process more efficient and smoother than the linear one does [28]. We describe the strength of the transverse field using following hyperbolic interpolation:

(9) $$\Gamma (t)=\frac{(1-t/\eta )\zeta }{t/\eta +\xi }$$

where *η* is the total annealing time and *ζ*, *ξ* are two control operators. This form is trivially Trotter-discretized [30], since the spin-flip term acts independently on the single spin at each time slot.

*(ii)*  Hamiltonian Approximation Based on Suzuki-Trotter Transformation

In order to figure out the OSTP selection problem by CPIMC-based QA, quantum Hamiltonian for the OSTP selection problem should be approximated by a classical one with the aid of a Suzuki-Trotter transformation. This is possible because of an analogy with a standard TISG model in a transverse field [22]. The transformation maps the quantum Hamiltonian to an effective classical Hamiltonian H [31], and then Equation (8) can be rewritten as:

(10) $$H=\frac{1}{P}\sum_{\rho =1}^{P}H_{\text{TISG}}(\{{ϒ}_{i,\rho }\})-J_{\Gamma }(\sum_{\rho =1}^{P-1}\sum_{i}{ϒ}_{i,\rho }{ϒ}_{i,\rho +1}+\sum_{i}{ϒ}_{i,1}{ϒ}_{i,P})$$

where *H* can be viewed as a consisting of *P* replicas $\left\{{ϒ}_{i,\rho },\rho =1,\;...,\text{​ }P\right\}$ of the classical potential energy of a given configuration $H_{pot}(\{{ϒ}_{i}\})$, with an interaction of a combined kinetic energy between them, ${ϒ}_{i,\rho }$ denotes the *i*th spin of the ρth replica. The term $J_{\Gamma }$ is the coupling among the replicas, which can be written as:

(11) $$J_{\Gamma }=-\frac{T}{2}\ln\tanh(\frac{\Gamma (t)}{PT})>0$$

where *T* is the temperature at which each replica is simulated.

### 3.4. Proposed QA_OSTP

In this part, we describe the components of our algorithm and the related key scheme design in detail.

As mentioned in Section 3.3, before the execution of QA_OSTP, all the values of trust between participants and recommendations of intermediate participants in wireless sensor networks should be obtained through mining techniques. The whole network structure is constructed from the recommendations from the intermediate participants. Then, this two-tier relationship information, together with the intermediate participants and these two QoT constraints, is treated as the input of the QA_OSTP algorithm, based on which the trustor node will be able to perform the algorithm. The output of QA_OSTP will be the *ground state* with the lowest energy in the related TISG model, which corresponds to the optimal social trust path between the trustor node and the target trustee node. The optimal social trust path should satisfy these two QoT-attribute constraints and yield the best utility with the criteria defined by the trustor node.

Notations that are used in QA_OSTP are shown in Table 2. Pseudo-code of QA_OSTP is given in Algorithm 1. The first outermost loop is controlled by *T*. Here, we choose a linear annealing schedule consisting of the initial temperature *T_0_* and *Max*_steps_. Each Monte Carlo step for QA_OSTP consists of a loop starting from the second outermost loop where *M* is a tunable multiplier; M×N moves are conducted at each step after which the control parameter is decreased. QA_OSTP keeps making the next Monte Carlo step each time until the termination condition is satisfied. The replicas are always connected to each other in numerical order in the same way throughout the search for spin products. The random disturbing means only changing the order in which replicas are selected for search. We find this is an efficient scheme, which can promote diversity in the population of configurations.

**Algorithm** **1.** Pseudo-code of QA_OSTP for WSNs.  Data: G, N,S_s_, D_s_, P, T_0_, Γ_0_,$V_{p}^{T_{•}}$,$V_{p}^{r_{•}}$, Max_steps_  Result: ostp_(Ss,…,Ds)_, $F_{ostp(S_{s},...,D_{s})}$ 1 begin 2 Set $T=T_{0}$,$\Gamma =\Gamma_{0}$,$\varpi =\{\omega_{\rho }\}$ 3 while T≠0 do 4   Randomly disturb the order of replicas 5   for φ=1,…,*P*, do 6    select the replica in position *φ* 7    for $N_{i}=1$,…M×N−1 do 8     Use “plus-minus” method to get ωρ' and hence ϖ 9     ΔH=H(ωρ')−H(ωρ) 10       if ΔH<0 then 11         ωρ=ωρ' 12       else 13         ωρ=ωρ' with probability exp(−ΔH/T) 14   end for 15  end for 16  $\Gamma =\Gamma -(\Gamma_{0}/Max_{steps})$ 17 end

## 4. Experimental Results

### 4.1. Experiment Setting

In our experiments, if not otherwise specified, all the related parameters are set following G. Liu. *et al.*’s work [11]. In order to evaluate our proposed algorithm, we compare QA_OSTP with MFPB_HOSTP and T-MONTE-K in terms of two key factors; that is, execution time and utility of the selected social trust path.

For QA_OSTP, we implement a similar CPIMC that was used in G. E. Santoro *et al.*’s work [26] at a fixed low temperature *T* (we used *T* = 10/3 K). The quantum model is mapped onto a classical model with an extra imaginary-time dimension, consisting of P ferromagnetically coupled replicas of the original spin problem, at temperature PT [26] (we used *P* = 30). Since QA requires initial configurations equilibrated at temperature PT, an obvious choice is to take PT = 100 [26]. Finally, the transverse field Γ is annealed hyperbolically in a MC time *τ* from an initial value Γ_0_ = 300 to a final value of zero. In QA, we used exclusively “plus-minus” tactic, with a static neighborhood pruning [32], which restricts the attempted neighborhood realization by allowing only a fixed number *M* (we used *M* = 20) of the nearest neighbors of participant *j* to be candidates for *j*’. Our MC step consisted of *M* × *N* attempted operations of “plus-minus” tactics (for QA, in each of the P replicas). In QA, we averaged the best social trust path utility found over up to 100 independent searches.

In our experiments, both two QoT parameters are randomly generated. The end-to-end QoT constraints specified by a trustor node are set as $V^{T}\geq 0.05$ and $V^{R}\geq 0.3$, respectively. We first randomly select 80 pairs of source and target participants from large scale WSNs with 87,474 nodes and 300,511 links. Moreover, following the small world and power-law characteristics, we set the maximal length of a social trust path to six hops. Then we number the different network scales from 1 to 25, with the number of nodes varying from 50 to 400 and number of links varying from 63 to 2356, respectively.

MFPB_HOSTP, T-MONTE-K, and our QA_OSTP were implemented in Matlab 7.0 and run on a PC with a 3 GHz Intel processor and 3 GB of RAM with Windows 7.

### 4.2. Performance Analysis

**Figure 3:**
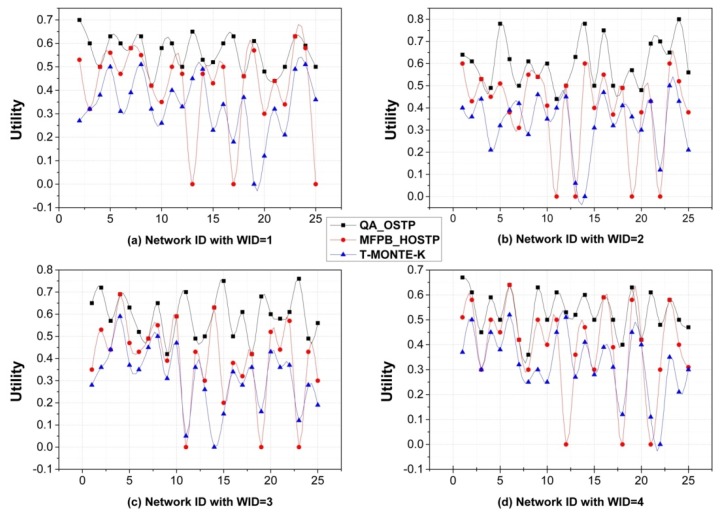
Comparison of path utilities of networks.

Figure 3 plots the utilities of the extracted social trust paths with different network scales and different weights of QoT constraints. The weights of QoT parameters are set as *w_T_* = 0.5, 0.75, 0.75, 0.6, *w_R_* = 0.5, 0.25, 0.25, 0.4, and each group corresponds to Weight IDs1, 2, 3, 4, respectively. From the figure, we could see that our QA_OSTP can always find utilities, each of which is not worse than that of MFPB_HOSTP and that of T-MONTE-K. This is because in QA_OSTP, quantum fluctuation is adopted to avoid the local minima and quantum mechanics works with wave functions that can sample different regions of phase space equally well. While for MFPB_HOSTP and T-MONTE-K, although sub-networks are extracted through exhaustive searching before the algorithm execution and Backward_Search scheme is used to estimate whether there exists a feasible solution in a sub-network, due to the intrinsic characteristic of heuristic algorithms, it does not guarantee that the best social trust path will be found. Moreover, with the network scale growing larger, when the social trust path with the maximal utility is not a feasible solution, the heuristic search can hardly find a near optimal solution and usually returns an infeasible one even when a feasible solution exists (e.g., case S3). Thus, in any case, QA_OSTP shows better performance. Specifically, we find that the mean value of utilities of QA_OSTP is 34.06% more than that of MFPB_HOSTP and 66.43% more than that of T-MONTE-K in Figure 3a; 50.65% and 78.10% more in Figure 3b; 50.05% and 89.14% more in Figure 3c; 35.82% and 62.32% more in Figure 3d.

**Figure 4:**
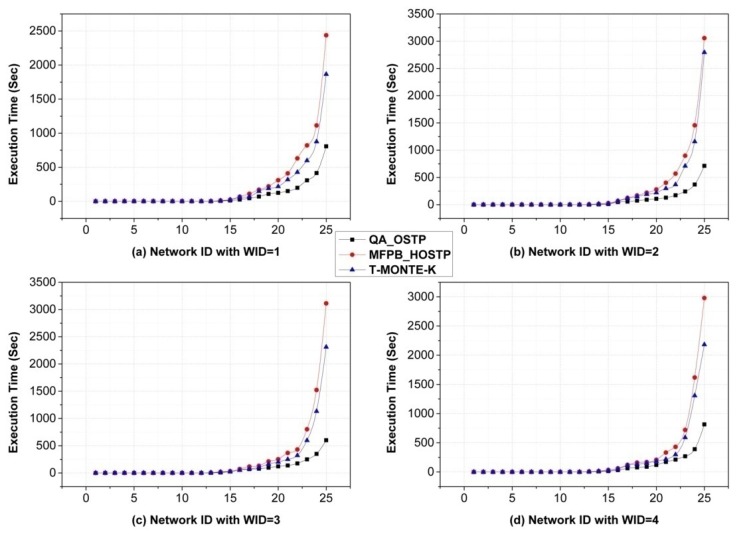
Comparison of execution time of algorithms.

Figure 4 shows the average execution time of algorithms with different weights of QoT parameters and different network scales. Note that, for MFPB_HOSTP and T-MONTE-K, execution time should include the exhaustive searching time for extracting sub-networks, which is not taken into consideration in G. Liu. *et al.*’s work. From Figure 4, we can see that when the network scale is not large, MFPB_HOSTP, T-MONTE-K, and QA_OSTP perform well and the difference is trivial because the searching space is relatively small. Nevertheless, with the network scale expanding, we can observe that QA_OSTP can outperform MFPB_HOSTP and T-MONTE-K in terms of execution time. This is an interesting result since annealing is a very slow physical process and may be a little time-consuming in statistical mechanics. Since the quantum Hamiltonian in QA_OSTP is approximated with the aid of a Suzuki–Trotter transformation in CPIMC-based QA, quantum tunneling can avoid some unnecessary searching in MFPB_HOSTP and T-MONTE-K, which can accelerate the annealing process. Specifically, the average execution time of our proposed algorithm is only 35.90% of that of MFPB_HOSTP and 47.38% of that of T-MONTE-K in Figure 4a; 27.80% and 33.23% in Figure 4b; 28.10% and 37.87% in Figure 4c; 33.19% and 43.16% in Figure 4d.

## 5. Conclusions

We have applied a quantum annealing-based algorithm, QA_OSTP, to solve the NP-complete OSTP selection problem in large scale WSNs, which is a first OSTP algorithm based on a novel configuration path-integral Monte Carlo (CPIMC) approach and inspired by quantum mechanics. Since the quantum annealing process has proven to be more effective than waiting for those barriers to be overcome thermally as in SA, QA_OSTP is able to find the configuration of comparable quality to the best algorithms. Thus, it shows that quantum annealing can be a very promising tool for solving the OSTP selection problem in WSNs. Based on the above experiment, we can observe that QA_OSTP is an effective and efficient algorithm, and it performs better than state-of-the-art heuristic algorithms, significantly, in terms of execution time and quality of the final configuration. In future work, we plan to further understand how quantum mechanics can quantitatively improve the solution of the OSTP selection problem and design a new trust oriented search engine where our proposed method will be applied to help a trustor node find the most trustworthy trustee node from all candidate nodes based on the given criteria in large scale WSNs.

## Figures and Tables

**Table 1:** Possible social trust path and corresponding state vector in Figure 2.

$U_{mn}$	Social Trust Path	State Vector	Utility
$U_{12}=1$ $\;U_{13}=0$	1—2—4	|01〉	0.616
$U_{12}=0$ $\;U_{13}=1$	1—3—4	|10〉	0.384
$U_{12}=1$ $\;U_{13}=1$	Invalid path	|11〉	Invalid
$U_{12}=0$ $\;U_{13}=0$	Invalid path	|00〉	Invalid

**Table 2:** Notations used in QA_OSTP.

*G*	Social graph of WSNs with two QoT parameters
*S*_s_	Trustor node
*D*_s_	Target trustee node
$F_{p}$	Utility of a social trust path configuration
*P*	The number of replicas
*T*	Temperature parameter
*T*_0_	Optimal initial temperature
*Γ*	Transverse field
*Γ*_0_	An initial value of the transverse field
*M*	Fixed number of nearest neighbors of participant
ρ	Sequence number of a certain replica
*N*	Number of nodes in WSNs
*φ*	The series number of replica
$\tau_{\text{total}}$	Total CPIMC time
*Max*_steps_	Maximum number of CPIMC steps
*N*_i_	Number of iterations
$\omega_{\rho }$	A social trust path configuration
ϖ	A series of $\omega_{\rho }$, also known as $\left\{\omega_{\rho }\right\}$
ωρ'	Neighbor of configuration $\omega_{\rho }$
$V_{p}^{T_{•}}$	Trust constraint of social trust path
$V_{p}^{r_{•}}$	Recommendation degree constraint of social trust path
